# Shortened Lifespan and Lethal Hemorrhage in a Hemophilia A Mouse Model

**DOI:** 10.1371/journal.pone.0154857

**Published:** 2016-05-04

**Authors:** Janice M. Staber, Molly J. Pollpeter

**Affiliations:** Stead Family Department of Pediatrics, Carver College of Medicine, University of Iowa, Iowa City, IA, 52242, United States of America; University of Pennsylvania School of Medicine, UNITED STATES

## Abstract

**Background:**

Hemophilia A animal models have helped advance our understanding of factor VIII deficiency. Previously, factor VIII deficient mouse models were reported to have a normal life span without spontaneous bleeds. However, the bleeding frequency and survival in these animals has not been thoroughly evaluated.

**Objective:**

To investigate the survival and lethal bleeding frequency in two strains of E-16 hemophilia A mice.

**Methods:**

We prospectively studied factor VIII deficient hemizygous affected males (n = 83) and homozygous affected females (n = 55) for survival and bleeding frequency. Animals were evaluated for presence and location of bleeds as potential cause of death.

**Results and Conclusions:**

Hemophilia A mice had a median survival of 254 days, which is significantly shortened compared to wild type controls (p < 0.0001). In addition, the hemophilia A mice experienced hemorrhage in several tissues. This previously-underappreciated shortened survival in the hemophilia A murine model provides new outcomes for investigation of therapeutics and also reflects the shortened lifespan of patients if left untreated.

## Introduction

Hemophilia mouse models have been available since the mid-1990s and have proven to be valuable tools for investigating many scientific questions [1, 2]. Bi *et al*. produced two strains of hemophilia A mice; one with a neo cassette disrupting exon 16 (E-16) of the factor VIII (FVIII) gene and one with a neo cassette disrupting exon 17 (E-17) [1]. Original descriptions indicated that E-16 FVIII knockout mice had a normal lifespan and no spontaneous bleeding phenotype [1, 2]. However, severe hemophilia A patients, if left untreated, have significant bleeding complications including joint bleeds and decreased survival with median life expectancy of 7.8 to 11 years [3, 4]. We previously observed that untreated E-16 FVIII deficient animals experienced decreased survival compared to animals receiving FVIII gene transfer [5]. Therefore, we investigated survival and bleeding frequency of E-16 FVIII null mice compared to wild type animals. In contrast to original reports, we observed that hemophilic mice on two different genetic backgrounds have spontaneous bleeds and a shortened lifespan. Importantly, this revised and previously under-recognized phenotype more accurately mirrors that of untreated hemophilia A patients and provides new endpoints when evaluating future therapeutics.

## Materials and Methods

All mice were housed under a 12:12 hour light-dark cycle at University of Iowa Animal Care Facilities under pathogen-free conditions for the duration of the study. Mice were housed in groups of as many as 5 mice per cage with a temperature set point of 70 degrees F. All animal procedures were approved by Institutional Animal Care and Use Committee at the University of Iowa. Hemophilia A mice with a targeted deletion of exon 16 were used in these studies [1, 5]. Two FVIII deficient mice strains were used including B6;129S-*F8*^*tm1Kaz*^ (originally purchased from Jackson Laboratories [Bar Harbor, ME] and the colony subsequently maintained with introduction of purchased breeder pairs approximately yearly) as well as congenic C57Bl/6J mice backcrossed for more than 7 generations [5]. The B6;129S-*F8*^*tm1Kaz*^ mice will be hence referred to as B6129 F8 null and the C57Bl/6J congenic FVIII deficient mice will be referred to as C57Bl/6 F8 null. Hemizygous affected males and homozygous affected females were studied. Both C57Bl/6J and B6129SF2/J wild type breeder pairs were purchased from Jackson Laboratories, and colonies were maintained with introduction of purchased C57Bl/6J or B6129SF2/J breeder pairs approximately yearly.

**Fig 1:**
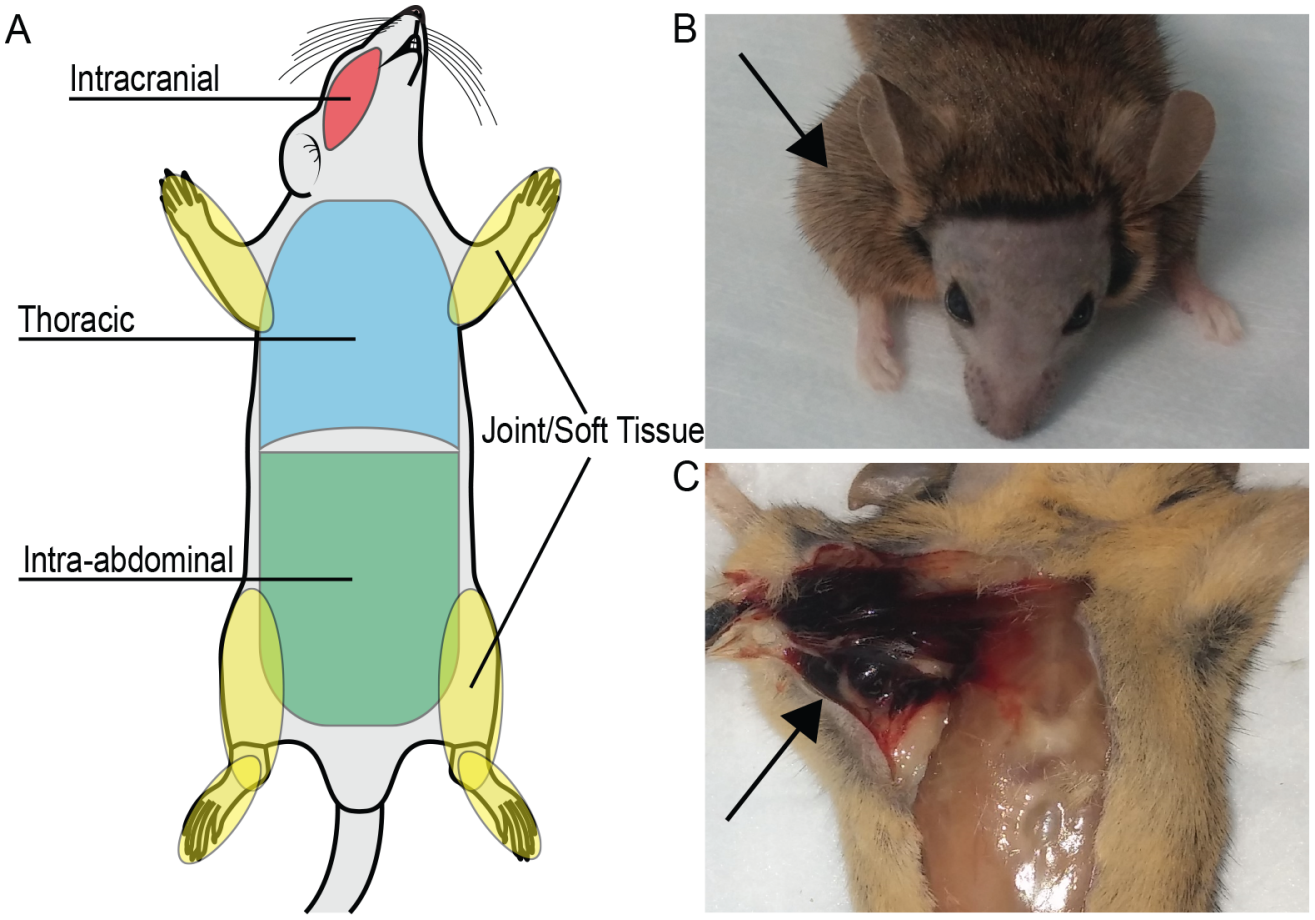
Hemophilia A mice sustain lethal bleeding events. **(A**) Illustration depicts sites of lethal bleeds which include intracranial, joint/soft tissue, thoracic, and intra-abdominal. **(B)** and **(C)** are representative pictures of gross bleeding observed during the study. **(B)** Mouse discovered dead in cage with swollen joint visibly noticeable (arrow indicates the affected joint). **(C)** Grossly visible joint/soft tissue bleed noted on necropsy (arrow indicates same joint as in (B)).

Interventions were limited to an ear punch for identification and retro-orbital bleed to collect whole blood as previously described [5]. No instruments were used during cage changing. Aggressive mice were separated to eliminate trauma-induced injury. Topical thrombin (King Pharmaceuticals, Bristol, TN) was applied as needed when minor superficial bleeds occurred. Cases of dermatitis were treated with triple antibiotic ointment. Mice were euthanized for the following reasons: limb weakness or paralysis, disinterest in eating or drinking, abnormal respirations, significant dermatitis not responding to adequate treatment, significant lethargy, or moribund appearance. If joint bleeding led to significant immobility resulting in the inability to independently obtain water and food, then mice were euthanized according to our institutional Animal Care Protocols. Time of euthanasia was taken as the best estimate for time of natural death, and euthanized mice were included in our statistical analysis. Otherwise, animals were inspected at least once daily to determine approximate date of death. At the time of death, necropsies were preformed to assess for grossly visible lethal bleeds. Animals were evaluated by a single investigator for presence and location of bleeds as potential cause of death. Cause of death was noted as non-bleeding, significant dermatitis (as deemed by the veterinarian), or lethal bleeding. Animals with lethal bleeds were further sub-classified by the site of bleed (Fig 1).

Whole blood was collected via retro-orbital bleed in sodium citrate at a final concentration of 0.38% (wt/vol) and plasma collected per laboratory protocol [5]. Factor VIII function was measured using a FVIII chromogenic assay (Chromogenix; Lexington, MA) as previously described [5]. Von Willebrand factor (vWF) antigen was measured via ELISA. Wells were pre-coated with a polyclonal anti-human vWF antibody (Dako, Carpinteria, CA) diluted 1:2,000. After blocking buffer, samples were allowed to incubate for 2 hours at 37°C. A horseradish peroxidase-conjugated polyclonal anti-human vWF antibody (Dako, Carpinteria, CA) diluted 1:10,000 was used for detection. A standard curve was generated from serial dilutions of normal pooled human plasma (George King Bio-Medical, Overland Park, KS) and antigen analyzed using the VersaMax Microplate Reader (Molecular Devies, Sunnyvale, CA) at wavelength 450nm.

EDTA-coated microcapillary tubes (JAS Diagnostics, Inc., Miami Lakes, FL) were used to collect whole blood. Samples were analyzed for platelet count and hematocrit using the ADVIA 120 Hematology System (Siemens, Erlangen, Germany).

Significant differences among groups were analyzed via the log-rank [Mantel-Cox] test. Analysis was performed in GraphPad Prism (GraphPad Software, La Jolla, CA). A *P* value of < 0.05 was considered statistically significant.

## Results and Discussion

All hemophilic mice demonstrated FVIII activity levels below the limit of detection (< 0.006 IU/mL) compared to wild type controls (0.63–1.23 IU/mL: average 0.881 IU/mL). Additionally, F8 null mice demonstrated normal platelet counts, hematocrits and von Willebrand factor (vWF) levels at approximately 4–6 weeks of age prior to any visible evidence of a bleeding event (Table 1).

**Table 1:** FVIII deficient mice have similar von Willebrand factor levels, platelet counts, and hematocrits compared to wild type mice.

	C57Bl/6	C57Bl/6	B6;129SF2/J	B6;129
		F8 null		F8 null
von Willebrand factor (% Ag)*	101.5 ± 44.1^§^	116.6 ± 48.6	88.2 ± 48.6	161 ± 109.1
Platelet count (x 10^9^/L)*	1158 ± 193.3	1170 ± 120.7	859.2 ± 86.6	814 ± 138.5
Hematocrit (%)*	50.6 ± 1.4	48.6 ± 1.8	52.6 ± 2.5	52.3 ± 5.7

* No significant difference between wild type and F8 null within each background (n = 5–8 per group)

^§^ Value ± standard deviation

**Fig 2:**
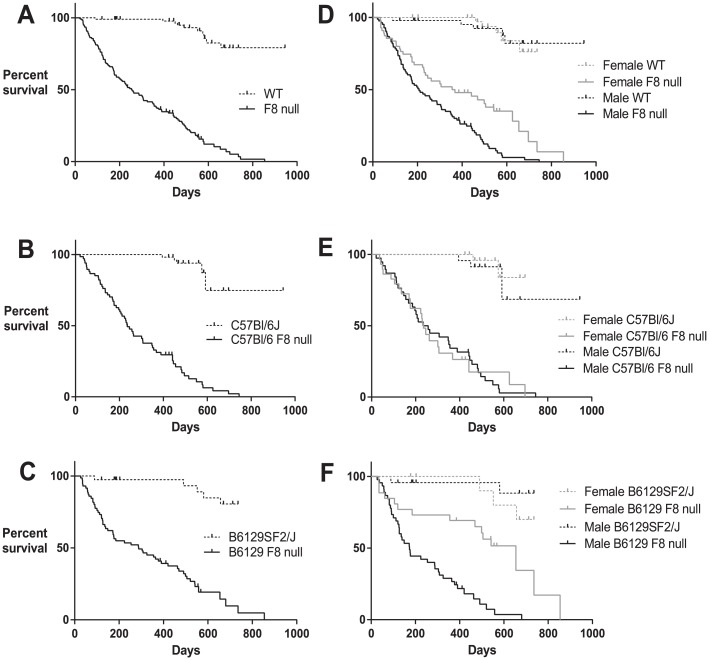
Hemophilia A mice have a shortened lifespan compared to wild type controls. **(A)** Hemophilia A mice have a median survival of 254 days. n = 138 (total from both backgrounds), p < 0.0001 (log-rank [Mantel-Cox] test) compared to wild type mice, n = 96. **(B)** C57Bl/6 F8 null mice have a median survival of 236 days. n = 67, p < 0.0001 (log-rank [Mantel-Cox] test) compared to wild type mice, n = 54. **(C)** B6129 F8 null mice have a median survival of 288 days. n = 71, p < 0.0001 (log-rank [Mantel-Cox] test) compared to wild type mice, n = 39. **(D)** Factor VIII deficient animals have decreased median lifespan regardless of gender. Median survival of F8 null female mice (361.5 days) was significantly longer than that of F8 null male mice (207 days). p = 0.0009, log-rank [Mantel-Cox] test. **(E)** Female C57Bl/6 F8 null mice have a median survival of 236 days, n = 29 (p < 0.0001 when compared to wild type controls, n = 31). Male C57Bl/6 F8 null mice have a median survival of 245.5 days, n = 38 (p < 0.0001 when compared to wild type controls, n = 23). **(F)** Female B6129 F8 null mice have a median survival of 654 days, n = 26 (p = 0.022 when compared to wild type controls, n = 16). Male B6129 F8 null mice have a median survival of 173 days, n = 45 (p < 0.0001 when compared to wild type controls, n = 23).

The median survival of the F8 null mice (n = 138, both backgrounds) was significantly lower when compared to wild type controls (n = 96, both backgrounds) (Fig 2A; p < 0.0001). To investigate if this difference was strain-dependent, we prospectively followed survival of two commonly used FVIII deficient mouse models, C57Bl/6J background as well as B6129SF2/J. The C57Bl/6 F8 null mice had a median lifespan of 236 days (Fig 2B; n = 67). The median lifespan of the B6129 F8 null mice was slightly longer at 288 days (Fig 2C; n = 71) although not significantly different from the C57Bl/6 F8 null mice (p = 0.16). Previous reports indicate the median lifespan of C57BL/6 is 866 days for females (n = 32) and 901 days for males (n = 32) [6].

To further investigate survival of FVIII deficient animals, we included both female and male affected mice. Median survival was significantly decreased in both male and female mice (Fig 2D; p < 0.0001) when compared to wild type controls. Median lifespan of all F8 null female mice (n = 55) was 361.5 days (Fig 2D; p < 0.0001 compared to wild type mice). The median lifespan of all F8 null male mice (n = 83) was 207 days (Fig 2D; p < 0.001 compared to wild type mice). Amongst F8 null animals, female median survival was longer than that of male animals (Fig 2D; p < 0.0001). However, a significant difference in survival between genders was only apparent in the B6129 F8 null mice (Fig 2F; p < 0.0001) and not in the C57Bl/6 F8 null mice (Fig 2E; p = 0.97). Even with our attempt to separate aggressive mice, it is possible that the difference in survival between female and male F8 null mice is due to trauma. However, we did not observe increased aggressive behavior in the B6129 strain compared to the C57Bl/6 strain. We do note that previous reports in other knockout models indicate that mouse strain can result in phenotypic differences between genders including survival [7]. Of note, the frequency of death due to hemorrhagic events in the B6129 F8 null female mice was similar to the frequency of death due to hemorrhagic events in the C57Bl/6 F8 null female mice (50% vs. 39%, respectively). Therefore, the difference in survival does not appear to be due to a difference in bleeding events. Overall, median lifespan of F8 null male and female mice was shortened compared to wild type controls regardless of strain background (Fig 2E and 2F).

To identify possible causes of the shortened lifespan, we investigated the lethal bleeding frequency in hemophilia A mice. A large percentage of the deaths in hemophilia A mice were due to lethal bleeding (51%, Table 2). Lethal hemorrhagic events included intracranial hemorrhage, thoracic hemorrhage, intra-abdominal hemorrhage such as bowel obstruction from a hematoma or splenic hemorrhage, and spontaneous joint bleeding with significant immobility (Fig 1). The increased mortality was not entirely explained by gross hemorrhage, therefore additional mortality in the FVIII deficient animals may be due to microscopic bleeding or an alternative mechanism of death.

**Table 2:** Occurrence of lethal bleeding in factor VIII deficient mice.

Site of bleeding	C57Bl/6 F8 null		B6129 F8 null		E-17 FVIII deficient^d^^[^^9^^]^	
	Male	Female	Male	Female	Male	Female
	N^b^ = 37	N = 23	N = 41	N = 16	%	%
Intra-abdominal	2 (6.45)^c^	5 (21.74)	6 (15.79)	1 (6.25)	3.7	1.70
Thoracic	5 (16.13)	1 (4.35)	7 (18.42)	2 (12.50)	12.1	5.40
Joint/Soft tissue	3 (9.68)	0	5 (13.16)	2 (12.50)	0.06	0
Intracranial	3 (9.68)	1 (4.35)	4 (10.53)	2 (12.50)	NR^e^	NR
Multiple sites	2 (6.45)	2 (8.70)	1 (2.63)	1 (6.25)	NR	NR
Non-bleed COD	15 (48.39)	14 (60.87)	15 (39.47)	8 (50)		
COD unknown^a^	7 (22.58)	0	3 (7.89)	0		

^a^ Unable to determine cause of death (COD), e.g. body cannibalized

^b^ N = total number of dead mice

^c^ Values expressed as number of mice (percentage of the total number of evaluable deaths)

^d^ Results reported from Muchitsch et al. 1999 [9]

^e^ NR—Not reported

A cause of death other than bleeding in the C57Bl/6 wild type colony included significant dermatitis leading to exposed lesions and/or exposed bone, and therefore animals were euthanized (n = 3). Ulcerative dermatitis was anticipated as the rate of dermatitis in our C57Bl/6 colony (5.6%) is consistent with that of previous reports (4.1%) [8] and none of our B6;129SF2/J mice developed dermatitis. Of note, significant dermatitis which did not improve occurred in the C57Bl/6 F8 null mice leading to euthanasia of 3 mice which are included as non-bleeding events in Table 2. Dermatitis was not observed in the B6129 F8 null mice.

Bleeding frequency leading to death has not been previously reported in the E-16 FVIII knockout mouse model. Bleeding at various sites previously reported in the E-17 FVIII knockout mice included a high frequency of lethal thoracic hemorrhage (12.1%) [9]. This is similar to our cohort of mice (average of 12.8%, Table 2). Our findings vary from that reported for the E-17 FVIII knockout, as our E-16 FVIII knockout mice had a higher rate of lethal intra-abdominal hemorrhage. E-17 FVIII knockout mice experienced a rate of 0.2% [9] compared to our average of 12.6% (Table 2). Despite separating aggressive mice, five hemophilia A mice had lethal injuries even when housed singly (most notably blood loss from toe injury). Additionally, our cohort of mice had an 8.5% average incidence of intracranial hemorrhage [10]. Importantly, no lethal bleeds were noted in the wild type colony. These data are consistent with both increased bleeding occurrence and poor wound healing defects in hemophilia patients [11].

Here we present new studies of survival and bleeding causes of death in FVIII null mice compared to wild type animals. From our data, a lethal bleeding phenotype is evident in the E-16 hemophilia A mouse model. Currently in patients with severe hemophilia A, the majority of deaths due to bleeding are the result of intracranial hemorrhage [10, 12]. We observed less than 10% of bleeding deaths as a result from intracranial hemorrhage which is less frequent than lethal hemorrhage from intracranial hemorrhage in hemophilia A patients. This may be due to different locomotion and/or activity of mice compared to humans.

A 1989 study evaluated the mortality and causes of death in hemophilia patients and indicated that bleeding occurred in half of all deaths [13]. Traumatic bleeding was the most common cause of lethal bleeding [13]. However, more recently, only 23% of deaths in severe hemophiliacs were related to hemorrhagic events [14], which is likely related to the use of factor replacement. The rate of lethal bleeds in our study was 51% which directly correlates to the hemophilic phenotype in humans. In contrast to original reports [1, 2], we observed that hemophilic mice on two different backgrounds have lethal bleeds and shortened lifespan which is strikingly consistent with that of untreated hemophilia A patients [3, 4]. These findings broaden our understanding of the phenotype of FVIII deficient mice and have important implications for the design and interpretation of therapeutic studies using these important models.

## References

[1] Bi L, Lawler AM, Antonarakis SE, High KA, Gearhart JD and Kazazian HH Jr. Targeted disruption of the mouse factor VIII gene produces a model of haemophilia A. Nature Genetics. 1995;10:119–21. 7647782 10.1038/ng0595-119

[2] Bi L, Sarkar R, Naas T, Lawler AM, Pain J, Shumaker SL, et al Further characterization of factor VIII-deficient mice created by gene targeting: RNA and protein studies. Blood. 1996;88:3446–50. 8896409

[3] Larsson SA. Life expectancy of Swedish haemophiliacs, 1831–1980. British J of Haematology. 1985;59:593–602. 10.1111/j.1365-2141.1985.tb07353.x 3885998

[4] Ikkala E, Helske T, Myllyla G, Nevanlinna HR, Pitkanen P and Rasi V. Changes in the life expectancy of patients with severe haemophilia A in Finland in 1930–79. Br J Haematol. 1982;52:7–12. 6810913 10.1111/j.1365-2141.1982.tb03856.x

[5] Staber JM, Pollpeter MJ, Arensdorf A, Sinn PL, Rutkowski DT and McCray PB. piggyBac-mediated phenotypic correction of factor VIII deficiency. Mol Ther Methods Clin Dev. 2014;1. 10.1038/mtm.2014.42 PMC4362371 26015980

[6] Yuan R, Tsaih SW, Petkova SB, Marin de Evsikova C, Xing S, Marion MA, et al Aging in inbred strains of mice: study design and interim report on median lifespans and circulating IGF1 levels. Aging cell. 2009;8:277–87. 10.1111/j.1474-9726.2009.00478.x 19627267 PMC2768517

[7] Shah S, Sanford UR, Vargas JC, Xu H, Groen A, Paulusma CC, et al Strain background modifies phenotypes in the ATP8B1-deficient mouse. PLoS One. 2010;5:e8984 10.1371/journal.pone.0008984 20126555 PMC2813882

[8] Kastenmayer RJ, Fain MA and Perdue KA. A retrospective study of idiopathic ulcerative dermatitis in mice with a C57BL/6 background. J Am Assoc Lab Anim Sci. 2006;45:8–12. 17089984

[9] Muchitsch EM, Turecek PL, Zimmermann K, Pichler L, Auer W, Richter G, et al Phenotypic expression of murine hemophilia. Thromb Haemost. 1999;82:1371–3. 10544939

[10] Larsson SA and Wiechel B. Deaths in Swedish hemophiliacs, 1957–1980. Acta Med Scand. 1983;214:199–206. 6660026 10.1111/j.0954-6820.1983.tb08595.x

[11] Sixma JJ and van den Berg A. The haemostatic plug in haemophilia A: a morphological study of haemostatic plug formation in bleeding time skin wounds of patients with severe haemophilia A. Br J Haematol. 1984;58:741–53. 6518139 10.1111/j.1365-2141.1984.tb06121.x

[12] Darby SC, Kan SW, Spooner RJ, Giangrande PL, Hill FG, Hay CR, et al Mortality rates, life expectancy, and causes of death in people with hemophilia A or B in the United Kingdom who were not infected with HIV. Blood. 2007;110:815–25. 17446349 10.1182/blood-2006-10-050435

[13] Rosendaal FR, Varekamp I, Smit C, Brocker-Vriends AH, van Dijck H, Vandenbroucke JP, et al Mortality and causes of death in Dutch haemophiliacs, 1973–86. Br J Haematol. 1989;71:71–6. 2917132 10.1111/j.1365-2141.1989.tb06277.x

[14] Lovdahl S, Henriksson KM, Baghaei F, Holmstrom M, Nilsson JA, Berntorp E, et al Incidence, mortality rates and causes of deaths in haemophilia patients in Sweden. Haemophilia. 2013;19:362–9. 10.1111/hae.12092 23374117

